# Supplementary material for: Collective self-caging of active filaments in virtual confinement
Source: Nat Commun. 2024 Oct 23;15:9122. doi: 10.1038/s41467-024-52936-9 (PMC11499643; doi:10.1038/s41467-024-52936-9)
Supplement: Supplementary file 1 — Supplementary Information [file 41467_2024_52936_MOESM1_ESM.pdf]

Collective self-caging of active filaments in virtual confinement

Maximilian Kurjahn,[1,*] Leila Abbaspour,[1,*] Franziska Papenfuß,[1] Philip Bittihn,[1]
Ramin Golestanian,[1,2] Benoît Mahault,[1,†] and Stefan Karpitschka[1,3,4,‡]

[1]*Max Planck Institute for Dynamics and Self-Organization (MPI-DS), 37077 Göttingen, Germany*
[2]*Rudolf Peierls Centre for Theoretical Physics, University of Oxford, OX1 3PU, Oxford, UK*
[3]*Fachbereich Physik, Universität Konstanz, 78457 Konstanz, Germany*
[4]*Centre for the Advanced Study of Collective Behaviour, Universität Konstanz, 78457 Konstanz, Germany*

(Dated: September 18, 2024)

Supplementary Figures

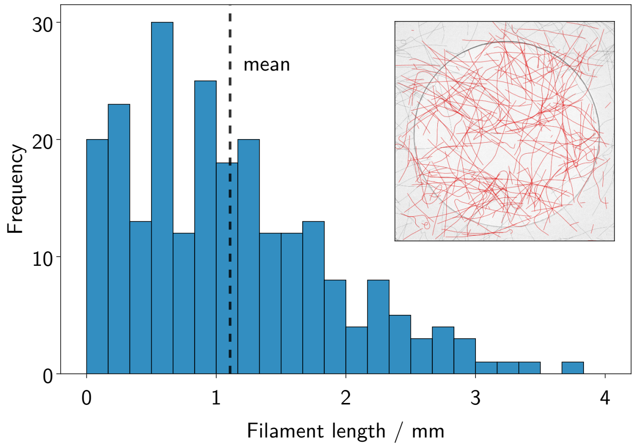

Fig. S1. **Statistics on filament length.** Length histogram of 238 manually measured filaments of the first snapshot (inset) of experiment shown in Fig. **1**(c) of the main manuscript. Only filaments that lie completely in the field of view are taken into account (red contours). Source data are provided as a Source Data file.

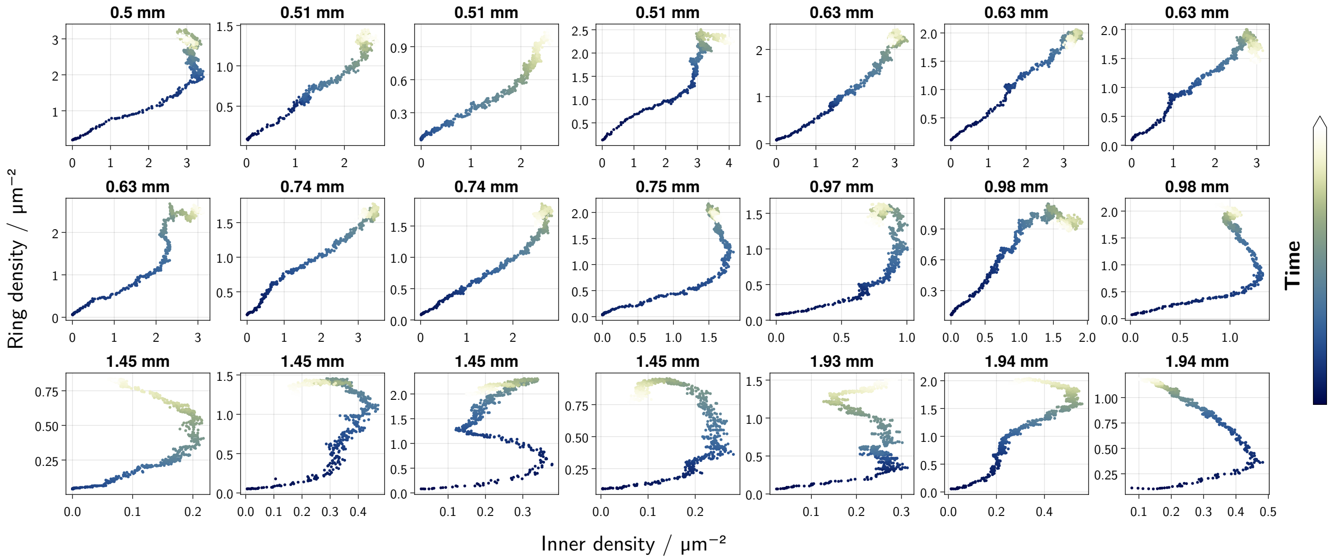

Fig. S2. **Critical density of ring formation for various radii** R**.** Inner mean density ($r < 0.8R$) against mean density of the ring ($0.8R < r < 1.1R$), where r is the radial coordinate and R the radius of the illumination mask. Panels are ordered by R. Time is color coded from the start of the experiment (dark blue) to the point of removing the mask (white). Source data are provided as a Source Data file.

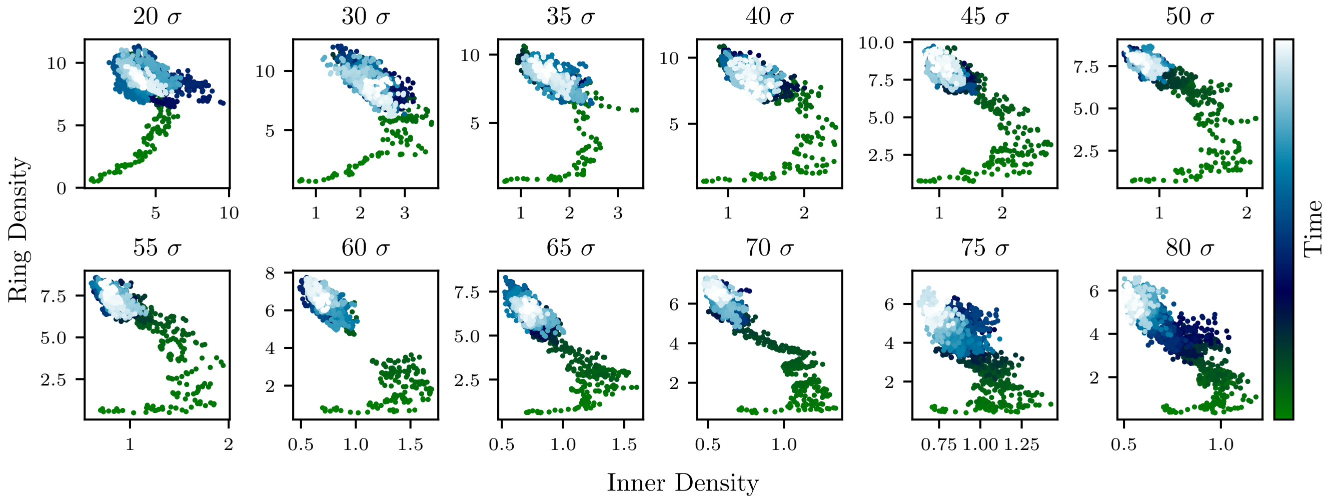

Fig. S3. **Critical density of ring formation for various radii** R **in simulations.** Inner mean density ($r < 0.8R$) against mean density of the ring ($0.8R < r < R$), where r is the radial coordinate and R the radius of the illumination mask. Panels are ordered by R , with its magnitude indicated above each panel. Time is color-coded from the start of the simulation (green) to the end of the simulation (light-blue). Source data are provided as a Source Data file.

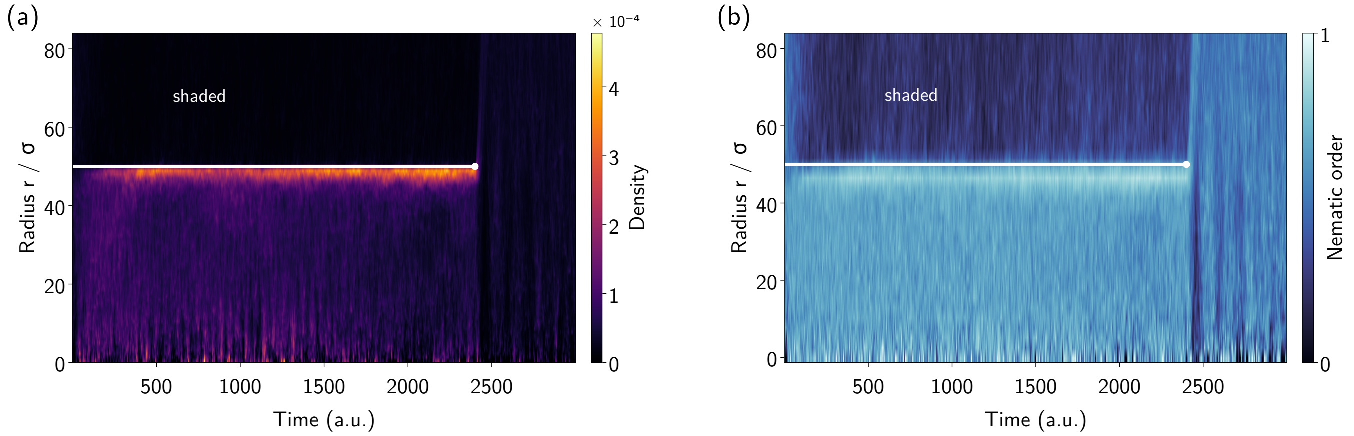

Fig. S4. **Kymographs of the simulation.** Azimuthally averaged (a) density and (b) nematic order as a function of time and radius of the simulation shown in Fig. **1**(e) of the main manuscript. The edge of the light patch is indicated by the horizontal white line and the removal of the light mask is indicated by the white dot at the end.

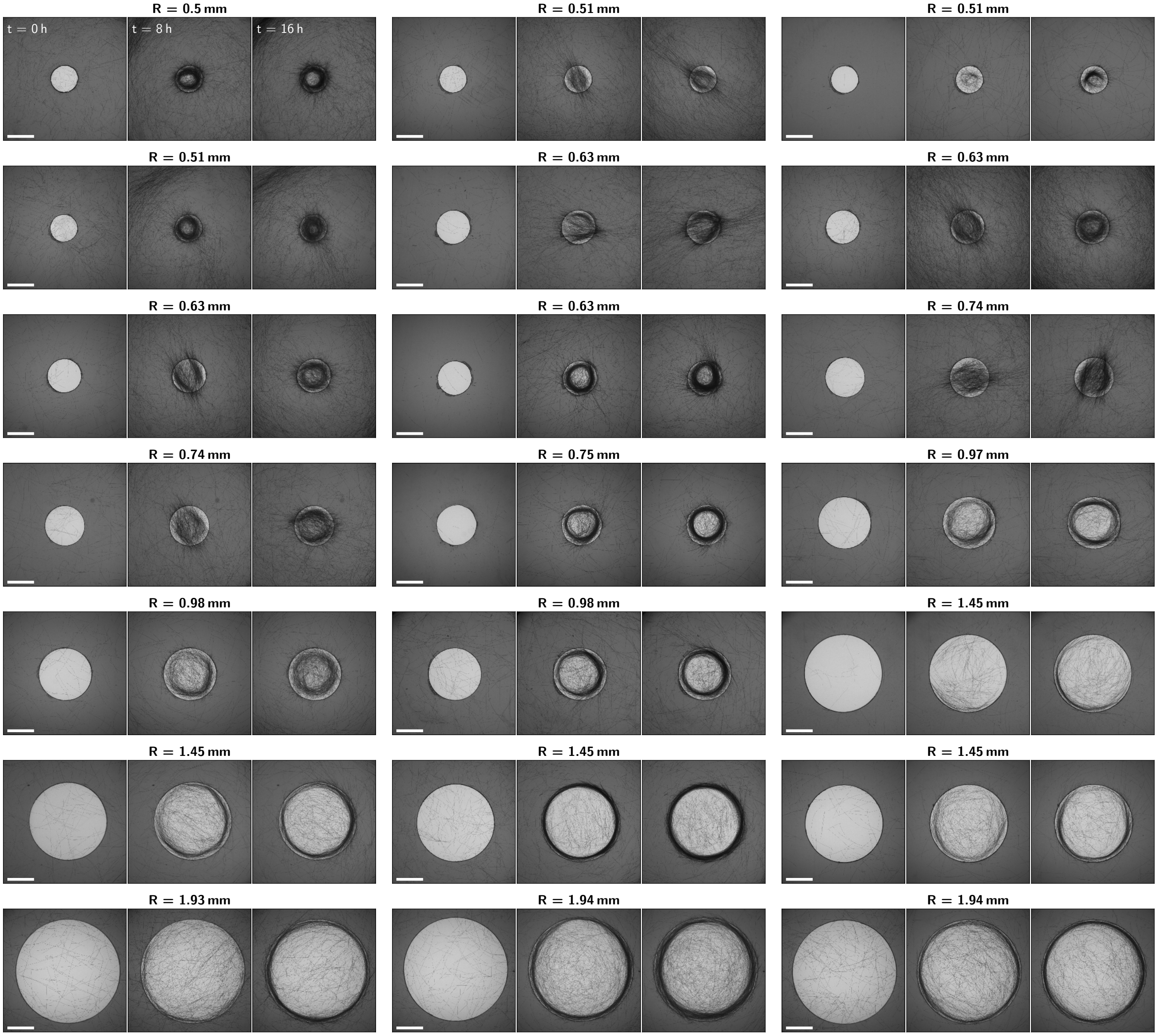

Fig. S5. **Local alignment to illumination boundary.** Experimental snapshots after $t = 0\,\mathrm{h}$, $8\,\mathrm{h}$, and $16\,\mathrm{h}$ for various illumination mask radii R. Panels are ordered by R. The scale bar is $1\,\mathrm{mm}$.

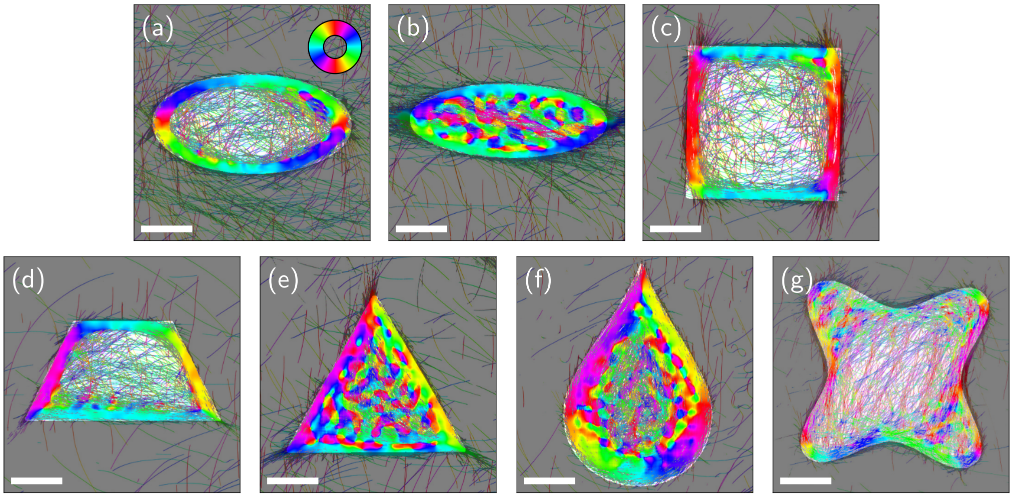

Fig. S6. **Local orientation for non-circular illumination.** (a)–(g) show the local orientation of stationary configurations experimentally obtained after ~ 17 to 21 hours of illumination, calculated with Eq. (4) of the main manuscript. The color code for the orientation is given in panel (a), scale bar is 1 mm.

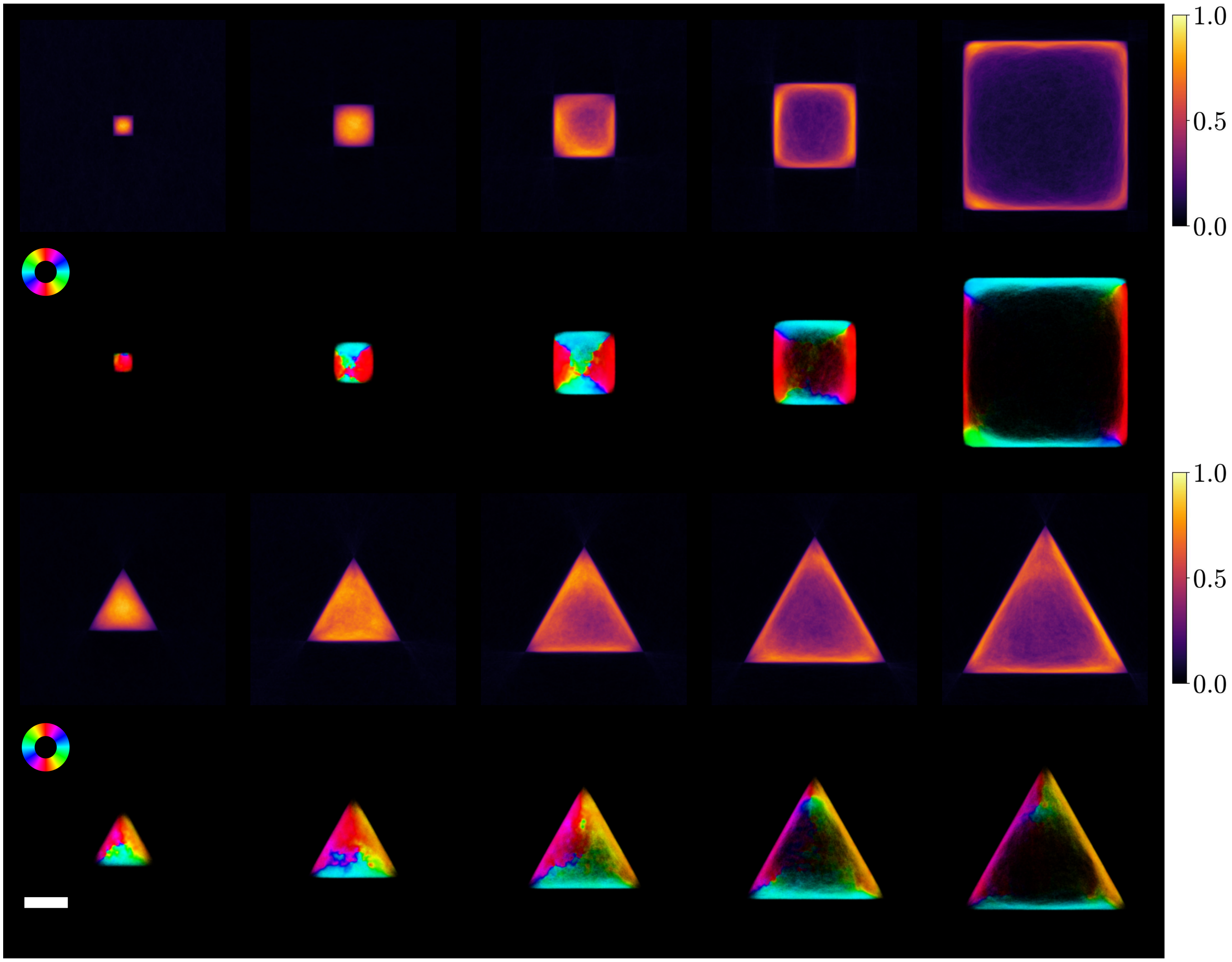

Fig. S7. **Simulations for polygons of different sizes.** Density (first and third rows) and nematic order (second and fourth rows) profiles obtained from numerical simulations of the active polymer model for square and triangular illumination patterns. As the size of the pattern decreases, boundary accumulation is progressively lost. The scale bar is 20σ.

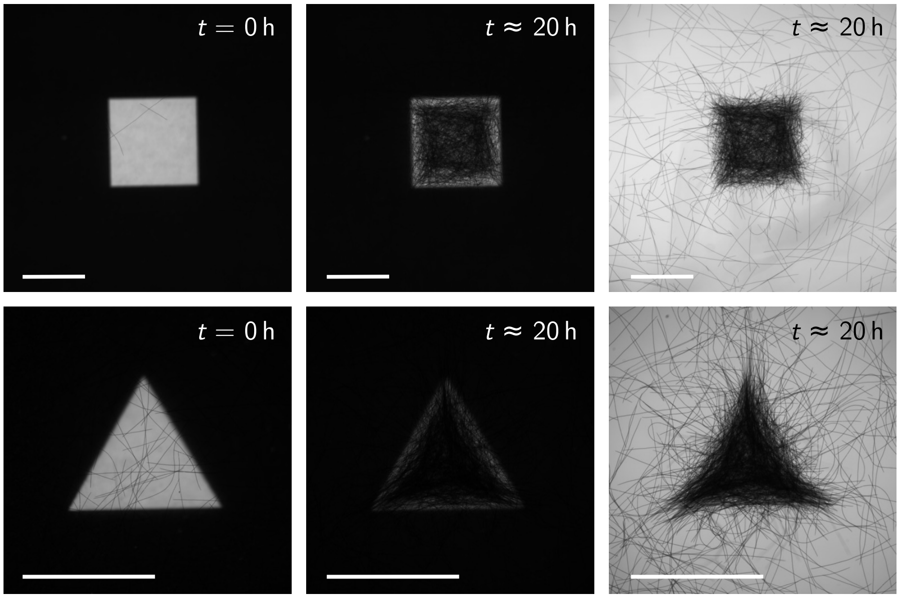

Fig. S8. **Experiments with small polygons.** Snapshots from experiments with small square (top) and triangle (bottom) illuminations spots, with sizes comparable to the typical filaments length (scale bar: 1 mm). The last two snapshots (middle and right) are obtained shortly before and after the mask is removed after $\sim$ 20 hours.

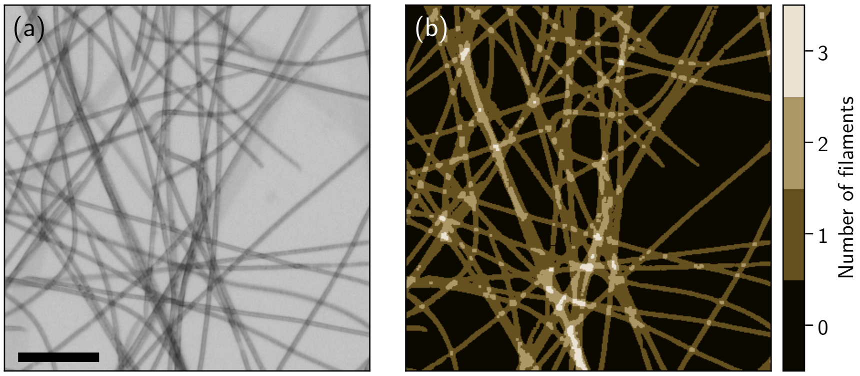

Fig. S9. **Local filament number density per pixel.** (a) Zoom into a micrograph of O. lutea, scale bar is 100 µm. (b) Number of overlapping filaments N per pixel calculated with Eq. (3) of the main manuscript and rounded to the next integer.

---

* M. K. and L. A. contributed equally to this work.
† E-mail: benoit.mahault@ds.mpg.de
‡ E-mail: stefan.karpitschka@uni-konstanz.de
