# Supplementary material for: Collective self-caging of active filaments in virtual confinement
Source: Nat Commun. 2024 Oct 23;15:9122. doi: 10.1038/s41467-024-52936-9 (PMC11499643; doi:10.1038/s41467-024-52936-9)
Supplement: Supplementary file 3 — Description of Additional Supplementary Files [file 41467_2024_52936_MOESM3_ESM.pdf]

Collective self-caging of active filaments in virtual confinement

Maximilian Kurjahn,[1,*] Leila Abbaspour,[1,*] Franziska Papenfuß,[1] Philip Bittihn,[1]
Ramin Golestanian,[1,2] Benoît Mahault,[1,†] and Stefan Karpitschka[1,3,4,‡]

[1]*Max Planck Institute for Dynamics and Self-Organization (MPI-DS), 37077 Göttingen, Germany*
[2]*Rudolf Peierls Centre for Theoretical Physics, University of Oxford, OX1 3PU, Oxford, UK*
[3]*Fachbereich Physik, Universität Konstanz, 78457 Konstanz, Germany*
[4]*Centre for the Advanced Study of Collective Behaviour, Universität Konstanz, 78457 Konstanz, Germany*

(Dated: October 7, 2024)

Description of Additional Supplementary Files

File Name: Supplementary Movie 1
Description: Scotophobic Response. Individual filaments of the cyanobacterium *O. lutea* reverse their gliding direction after entering a dark region. The dark region was generated by shading the transmission illumination of the microscope by a green filter that blocks wavelengths at which *O. lutea* primarily absorbs light. The scotophobic response manifests as a reversal of the gliding direction by 180°, rendering the motion purely one-dimensional. No active re-orientation occurs. The movie has been accelerated 120 times.

File Name: Supplementary Movie 2
Description: Emergent alignment and accumulation at the boundary of a light patch. Ensembles of the filamentous cyanobacterium *O. lutea* glide across a solid surface, aligning with and accumulating at the edge of a light spot. Individuals are not capable of active re-orientation, which emerges only as a collective effect. After removing the mask, the boundary accumulation dissolves. The movie has been accelerated 1440 times (real time in hh:mmm is given in the top-right corner).

File Name: Supplementary Movie 3
Description: Ring formation in the active polymer model. Each filament is colored according to its orientation (legend given in the top right corner). As in the experiments, the filaments can cross each other and do not reorient when they meet the edge of the illuminated area (white disk). After a brief transient, they self-assemble collectively into a ring structure characterized by a higher filament density at and a local nematic order along the illuminated domain. After removing the mask during the last 1/5 of the simulation time, the boundary accumulation dissolves. The simulation parameters are given in Table 1 of “Methods”.

File Name: Supplementary Movie 4
Description: Absence of ring formation in strictly 2D confinement. For an interaction range $d = 0.5\sigma$, the filaments cannot cross each other when they meet, which leads to the formation of highly dynamic traveling clusters that prevent the formation of a ring. Other simulation parameters are given in Table 1 of “Methods”.

---

* M. K. and L. A. contributed equally to this work.
† E-mail: benoit.mahault@ds.mpg.de
‡ E-mail: stefan.karpitschka@uni-konstanz.de
